# Serum Creatinine and Serum Cystatin C are Both Relevant Renal Markers to Estimate Vancomycin Clearance in Critically Ill Neonates

**DOI:** 10.3389/fphar.2021.634686

**Published:** 2021-03-19

**Authors:** Stéphanie Leroux, Valérie Biran, John van den Anker, Verena Gotta, Wei Zhao, Daolun Zhang, Evelyne Jacqz-Aigrain, Marc Pfister

**Affiliations:** ^1^ Department of Pediatrics/Neonatology, CIC 1414, CHU Rennes, Rennes, France; ^2^ Pediatric Pharmacology and Pharmacometrics, University Children’s Hospital Basel, University of Basel, Basel, Switzerland; ^3^ Department of Pediatric Pharmacology and Pharmacogenetics, Robert Debré Hospital, Paris, France; ^4^ Neonatal Intensive Care Unit, Robert Debré Hospital, Paris, France; ^5^ Division of Clinical Pharmacology, Children's National Hospital, Washington, D.C., WA, United States; ^6^ Department of Clinical Pharmacy, School of Pharmaceutical Sciences, Shandong University, Jinan, China

**Keywords:** serum cystatin C, serum creatinine, population pharmacokinetics, vancomycin, neonates

## Abstract

**Purpose:** Serum creatinine (SCr) is used as a marker of kidney function to guide dosing of renally eliminated drugs. Serum Cystatin C (S-CysC) has been suggested as a more reliable kidney marker than SCr in adults and children. Purpose of this study was to investigate S-CysC as alternative renal marker to SCr for estimating vancomycin clearance in neonates undergoing intensive care.

**Methods:** Vancomycin pharmacokinetics (PK), SCr and S-CysC data were collected in patients undergoing vancomycin treatment in the neonatal intensive care unit of Robert Debré Hospital - Paris. A population PK analysis was performed utilizing routine therapeutic drug monitoring samples. S-CysC and SCr were compared as covariates on vancomycin clearance using stepwise covariate modeling (forward inclusion [*p* < 0.05] and backward elimination [*p* < 0.01]). Model performance was evaluated by graphical and statistical criteria.

**Results:** A total of 108 vancomycin concentrations from 66 patients (postmenstrual age [PMA] of 26–46 weeks) were modeled with an allometric one-compartment model. The median (range) values for SCr and S-CysC were 41 (12–153) µmol/l and 1.43 (0.95–2.83) mg/l, respectively. Following stepwise covariate model building, SCr was retained as single marker of kidney function (after accounting for weight and PMA) in the final model. Compared to the final model based on SCr, the alternative model based on S-CysC showed very similar performance (e.g. BIC of 578.3 vs. 576.4) but included one additional covariate: impact of mechanical ventilation on vancomycin clearance, in addition to the effects of size and maturation.

**Conclusion:** ill neonates. However, if using S-CysC for this purpose mechanical ventilation needs to be taken into account.

## Introduction

Kidney function is a major determinant of clearance (CL) of renally eliminated drugs. Markers reflecting kidney function are therefore essential for dosing individualization. Utilizing optimal renal markers for optimizing drug dosing is a current pharmacological challenge in neonatal medicine.

Serum Creatinine (SCr) is used as a marker of glomerular filtration rate (GFR) to guide dosing of drugs eliminated by the kidney across all age groups. However, the use of SCr for this purpose raises two specific issues in neonates. First, SCr concentrations in the first days after birth are influenced by maternal creatinine, because of placenta transfer (Kastl, 2017). Second, SCr may be falsely elevated in the first days to weeks of life in premature neonates, because of tubular reabsorption by the immature kidney (Kastl, 2017).

Serum Cystatin C (S-CysC) has been suggested as a more reliable biomarker than SCr for monitoring kidney function in adults and children (Dharnidharka et al., 2002; Björk et al., 2019). In neonates, S-CysC offers the advantage of limited ability to cross the placental barrier (Allegaert et al., 2015). However, there is limited pharmacokinetic evidence for the usefulness of S-CysC as covariate explaining inter-individual variability of drug renal CL in neonates, especially in those undergoing intensive care treatments.

As vancomycin is one of the drugs primarily eliminated by glomerular filtration, and also highly prescribed in neonates, this glycopeptide antibiotic was used as model drug to answer our research question. The purpose of this pharmacokinetic (PK) study was to investigate S-CysC as alternative renal marker to SCr for explaining inter-individual variability of vancomycin CL in a representative cohort of neonates undergoing intensive care treatment.

## Materials and Methods

### Study Population and Design

This study was conducted in the neonatal intensive care unit (NICU) of Robert Debré University Hospital—Paris (France). Neonates receiving intravenous vancomycin as part of their routine clinical care were enrolled. Vancomycin dosing followed the individualized local guidelines routinely used in the unit (Zhao et al., 2013). A loading dose was infused over 60 min and followed by a maintenance dose administered as a continuous infusion over 24 h.

Vancomycin routine therapeutic drug monitoring (TDM) samples were used for PK modeling. The following data were collected and evaluated as covariates with a potential influence on vancomycin PK: gestational age (GA), postnatal age (PNA), postmenstrual age (PMA, defined as the sum of GA and PNA), birth weight (BW), current weight (CW), SCr and c-reactive protein (CRP) concentrations (both collected within 48 h of TDM sampling), mechanical ventilation, and co-medication of vasopressor agents or aminoglycosides. Patients with missing SCr data were excluded from analysis. Serum Cystatin C concentrations were measured on blood samples remaining after routine vancomycin TDM analysis which had been kept frozen at ˗ 80°C (maximum storage period of 12 months). No additional blood volume was taken for this non-interventional study. This study was approved by the ethics committee of Robert Debré University Hospital.

### Assay of Serum Vancomycin, Creatinine and Cystatin C

Serum vancomycin concentrations were measured by fluorescence polarization immunoassay using a Cobas Integra system (Roche Diagnostics, Meylan, France). The lower limit of quantification for this assay was 0.74 mg/l.

SCr concentrations were determined by an enzymatic method using the Advia 1800 chemistry system (Siemens Medical Solutions Diagnostics, Puteaux, France). The lower limit of quantification was 13 μmol l^−1^.

S-CysC concentrations were measured by an immunoenzymatic method using the Gentian AS (Moss, Norway) on Beckman Coulter (Beckman Coulter SA, Villepinte, Roissy CDG France). The Gentian Cystatin C immunoassay is a Particle-Enhanced Turbidimetric Immunoassay (PETIA). The lower limit of quantification was 0.4 mg/l.

All assays were performed in Robert Debré University Hospital, Paris.

### Population Pharmacokinetic Modeling of Vancomycin

#### Model Building

PK data for vancomycin were modeled with the software package NONMEM; parameters were estimated by first-order conditional estimation with interaction (FOCE-I).

As a first step, a structural model (without covariates) was developed. One- and two-compartment models were tested. As a second step, a stepwise covariate model building was performed applying a forward selection and backward elimination method (Mandema et al., 1992). The likelihood ratio test was used to test the effect of each covariate on model parameters. Power, exponential and linear models’ functions were tested to describe covariate effects of continuous variables. In addition, effect of PMA on CL was tested by means of a sigmoidal maximum effect function (Wang et al., 2019). During the initial step of covariate model building, inclusion of a covariate required a significant (*p* < 0.05; Likelihood ratio test) decrease in the objective function value (OFV; reduction >3.84 according to Chi-square distribution with one degree of freedom) from the basic model and a concomitant reduction in inter-individual variability (IIV) of the PK parameter. All of the significant covariates were then added simultaneously into an intermediate full model, starting with the most significant. Subsequently, each covariate was independently removed from the full model. Covariates were retained in the final model if a significant (*p* < 0.01; Likelihood ratio test) increase (more than 6.635 points) of the OFV was achieved.

As previously described in the literature for primarily renally eliminated antibiotics in neonates, vancomycin CL was finally parameterized as follows (Wilbaux et al., 2016):

CL = CLstandard * Effsize * Effmaturation * Effkidney

where CL standard represents the typical value of clearance in the study population, Effsize represents the effect of growth (modeled using allometric scaling approach), Effage represents the effect of age-dependent maturation (antenatal development and postnatal maturation being modeled using maturation functions including either PMA alone, or a combination of GA and PNA, or a combination of BW [as surrogate of GA] and PNA (Grubb et al., 2005), and Effkidney represents the effect of kidney function.

Reflecting the effect of kidney function, SCr, S-CysC and three preexisting estimates of GFR (Filler and Lepage, 2003; Grubb et al., 2005; Zappitelli et al., 2006) were compared as covariates on vancomycin CL.

#### Model Evaluation

The performance of developed model was assessed by graphical and statistical criteria. Goodness-of-fit plots were initially used for diagnostic purpose. The stability of the final model was also assessed using a nonparametric bootstrap analysis (Parke and Charles, 2000) with resampling and replacement (500 times). Values of estimated parameters obtained with the bootstrap procedure were compared with respective values estimated with original data set. Then, the final model was evaluated by calculating normalized prediction distribution errors (NPDE) (1,000 datasets were simulated with the final population model parameters) (Comets et al., 2008).

### Further Exploration of Differences Between SCr and S-CysC in Respect to Study Purpose

In order to investigate more in depth the relationship vancomycin CL - S-CysC to the relationship vancomycin CL - SCr, two vancomycin PK models were compared: one based on S-CysC (CYS model) and the other based on SCr (CR model). The Bayesian information criterion (BIC) was used to compare these non-nested models (Mould and Upton, 2013).

Additionally, in order to compare the behavior of both renal markers in our population with previous findings from the available literature, we explored effects of age and aminoglycosides on SCr and S-CysC levels. At this step, differences between two groups were assessed by the non-parametric Mann–Whitney–Wilcoxon test. Normality of the distributions was previously analyzed with the Shapiro–Wilk test. Statistical analyses were conducted using R software. A *p* value of <0.05 was considered statistically significant.

## Results

### Patient Characteristics

A total of 108 serum vancomycin concentrations from 66 patients were available for PK analysis, after exclusion of two patients because of lack of SCr data. Blood samples were drawn at a median of 33 h (range 5–354 h) after initiation of treatment. One to five PK samples per patient were available for analysis. Vancomycin concentrations ranged from 7.3 to 63.6 mg/l (Supplementary Figure S1). Baseline patient characteristics are presented in Table 1. GA and PMA ranged from 23 to 41 weeks and from 26 to 46 weeks, respectively. The median SCr and S-CysC concentrations were 41 µmol/l and 1.43 mg/l, respectively. Among the 66 patients, 70% had CRP concentrations >10 mg/l, 48% received mechanical ventilation and 23% were also treated with vasopressor agents at time of first vancomycin dosing.

**TABLE 1:** Baseline characteristics of the 66 patients.

	No. of neonates	Median (range)
Demographic data^a^		
Gender (female/male)	37/29	
Gestational age (weeks)		32 (23–41)
Postnatal age (days)		13 (1–106)
Postmenstrual age (weeks)		34 (26–46)
Birth weight (grams)		1590 (512–3950)
Current weight (grams)		1925 (530–3840)
Clinical data^a^		
Mechanical ventilation	32	
Coadministration of vasopressor agent(s)	15	
Coadministration of aminoglycoside(s)	39	
Biological data^b^		
Serum Creatinine concentration (µmol/L)		41 (12–153)
Serum Cystatin C concentration (mg/L)		1.43 (0.95–2.83)
C-reactive protein concentration (mg/L)		30 (5–313)
Vancomycin treatment data		
Loading dose (mg/kg)		10.5 (7.4–20.9)
First maintenance dose (mg/kg/day)		24.8 (10.3–59.3)

^a^ At time of first dosing

^b^ Within 48 h of first dosing

### Model Building

Data were best fitted by a one-compartment model with first-order elimination. Inter-individual variability could be estimated only for CL (exponential model). A proportional model best described the residual unexplained variability.

The allometric size approach, which consisted of a priori incorporation of CW into the structural model, generated a significant drop in the OFV (−50 units). Allometric exponents of 0.75 and 1 were fixed for CL and volume of distribution, respectively (Holford, 1996); their estimation did not improve the fit of the data. When tested individually, among all above-mentioned tested covariates, the following led to a significant decrease in the OFV from the allometric model: PMA, SCr, S-CysC, mechanical ventilation and co-medication of vasopressor agents. The results of the covariate analysis are presented in Table 2. Reflecting the maturation effect, PMA was superior as a covariate on CL, over the combination of BW and PNA and the combination of GA and PNA. Reflecting the effect of kidney function, SCr was superior as a covariate on CL over S-CysC and over all three tested estimates of GFR (Parke and Charles, 2000; Filler and Lepage, 2003; Zappitelli et al., 2006). Inclusion of SCr and S-CysC into the allometric model reduced inter-individual variability of vancomycin clearance (IIVCL) from 47 to 34% and 40%, respectively. Relationships between covariates and vancomycin CL were best fitted by power models functions for both SCr and S-CysC.

**TABLE 2:** Summary of covariate analysis^h^.

Characteristics	Pharmacokinetic parameters	OFV^e^	ΔOFV^f^	IIVCL^g^ (%)
Structural model	/	**644.8**	/	**71.0**
Allometric model (effect of size)	**CL, V**			
**CW**		**594.4**	**−50.4**	**47.3**
Effect of maturation^a^	**CL**			
BW and PNA		576.3	−68.5	40.1
GA and PNA		572.8	−72.0	40.0
**PMA**		**572.6**	**−72.2**	**40.0**
Effect of kidney function^a^	**CL**			
Grubb eGFR^b^		604.6	−40.2	41.5
Filler eGFR^c^		599.4	−45.4	41.2
Zappitelli eGFR^d^		599.1	−45.7	41.2
S-CysC		577.2	−67.6	40.2
**SCr**		**560.3**	**−84.5**	**33.8**
Effect of mechanical ventilation^a^	**CL**			
Ventilation		584.9	−59.9	41.8
Effect of maturation and kidney function^a^	**CL**			
**PMA and SCr (Final model)**		**548.3**	**−96.5**	**30.9**
PMA and SCr and S-CysC		544.6	−100.2	30.6
Effect of maturation, kidney function and mechanical ventilation^a^	**CL**			
PMA, SCr and ventilation		546.3	−98.5	29.9

CW current body weight; BW birth body weight; PNA postnatal age; GA gestational age; PMA postmenstrual age; eGFR estimate of glomerular filtration rate; S-CysC serum Cystatin C concentration; SCr serum Creatinine concentration; CL clearance, V volume of distribution

^a^ Included into the allometric model

^b^ eGFR = 84.69 × (S-CysC)-1.680 × 1.384if < 14 yr (Grubb et al., 2005)

^c^ eGFR = 91.62 × (S-CysC)-1.123 (Filler and Lepage, 2003)

^d^ eGFR = 75.94 × (S-CysC)1.17 × 1.2if renal transplant (Zappitelli et al., 2006)

^e^ Objective function value

^f^ Variation in objective function value

^g^ Inter-individual variability of vancomycin clearance

^h^ The characteristics in boldface were retained in the final population model.

After integration of SCr into the allometric model, PMA caused a further significant drop in the OFV (˗12 units). Other covariates were then rejected during the forward selection step because of insufficient decrease of the OFV. In particular, further introduction of S-CysC did not significantly improve the model (drop in the OFV of 3.7 units). The final model (CR model) included CW, PMA, and SCr as significant covariates on vancomycin CL (Table 3).

**TABLE 3:** Final estimates of population pharmacokinetic parameters of vancomycin and bootstrap results—from the final model based on Serum Creatinine (CR model).

Parameters	Final model		Bootstrap (*n* = 500)		
	Mean estimate	RSE (%)	Median	5th %ile	95th %ile
CL (L/h)					
CL = CLTV × (CW/1925)0.75 × Effage × Effkidney					
CLTV	0.11	4.4	0.11	0.10	0.12
Effage = (PMA/34)k1					
k1	1.47	23.3	1.51	0.94	2.12
Effkidney = (1/(SCR/41))k2					
k2	0.50	18.3	0.51	0.34	0.65
V (L)					
V = VTV × (CW/1925)					
VTV	0.47	44.8	0.48	0.14	1.30
Inter-individual variability (%)					
CL	30.9	27.3	29.8	23.0	36.6
Residual variability (%)	29.8	18.1	28.4	22.0	33.5

CL clearance; CLTV typical value of clearance; CW current body weight (grams); Effage effect of age; Effkidney effect of kidney function; PMA postmenstrual age (weeks); SCR Serum Creatinine concentration (µmol/L); V volume of distribution; VTV typical value of volume of distribution.

### Model Evaluation

No major bias in the goodness-of-fit plots was observed (Supplementary Figure S2). The median parameter estimates resulting from the bootstrap procedure closely agreed with the respective values from the final model (Table 3). The mean and variance of NPDE were 0.03 (Wilcoxon signed rank test *p* = 0.89) and 1.05 (Fisher variance test *p* = 0.70), respectively.

### Further Exploration of Differences Between SCr and S-CysC in Respect to Study Purpose

When S-CysC was selected instead of SCr as biomarker reflecting kidney function, the covariate selection process led to an alternative vancomycin PK model including CW, PMA, S-CysC and mechanical ventilation as covariates on CL (CYS model, Table 4). After integration of PMA and S-CysC into the allometric model, mechanical ventilation (associated with 30% reduced vancomycin CL) caused a further significant drop of 14 units in the OFV and a further reduction in IIVCL from 36.1 to 30.3%.

**TABLE 4:** Final estimates of population pharmacokinetic parameters of vancomycin and bootstrap results—from the alternative model based on Serum Cystatin C (CYS model).

Parameters	Final model		Bootstrap (*n* = 500)		
	Mean estimate	RSE (%)	Median	5th %ile	95th %ile
CL (L/h)					
CL = CLTV × (CW/1925)0.75 × Effage × Effkidney × Effventilation					
CLTV	0.15	6.2	0.14	0.13	0.16
Effage = (PMA/34)k1					
k1	1.72	19.7	1.76	1.17	2.42
Effkidney = (1/(SCYS/1.43))k2					
k2	0.97	20.1	0.98	0.59	1.31
Effventilation = k3					
k3	0.69	9.5	0.69	0.58	0.81
V (L)					
V = VTV × (CW/1925)					
VTV	0.48	40.3	0.48	0.14	1.22
Inter-individual variability (%)					
CL	30.3	24.6	29.2	22.2	34.9
Residual variability (%)	29.6	21.1	28.1	20.8	33.6

CL clearance; CLTV typical value of clearance; CW current body weight (grams); Effage effect of age; Effkidney effect of kidney function; Effventilation effect of mechanical ventilation; k3 scaling factor applied for patients receiving mechanical ventilation; PMA postmenstrual age (weeks); SCYS Serum Cystatin C concentration (mg/L); V volume of distribution; VTV typical value of volume of distribution.

The typical estimated values of vancomycin CL were very similar between CR and CYS models (i.e. 0.11 and 0.15 L/h for the CR and CYS models, respectively, with <10% relative standard errors of CL for both models). CL estimates (mean ± SD) obtained from CR and CYS model were 0.068 ± 0.025 l/h kg and 0.070 ± 0.025 l/h kg, respectively. Table 5 shows the high similarity between individual vancomycin CL estimates from CR and CYS models for four typical patients with different PMA. Both CR and CYS models showed very similar performance in terms of visual goodness of fit (Supplementary Figures S2 and S3), stability (Tables 3 and 4), NPDE results (for CYS model, the mean and variance of NPDE were 0.01 [*p* = 0.95] and 1.05 [*p* = 0.69], respectively), and BICs values (i.e. 576.4 and 578.3 for the CR and CYS models, respectively). To summarize, compared to the final model based on SCr, the alternative model based on S-CysC provided a very similar fit of the data but included the effect of mechanical ventilation on vancomycin CL as additional covariate.

**TABLE 5:** Individual vancomycin clearance estimates from CR and CYS models for four typical patients (1,000 simulations).

Postmenstrual age (weeks)	Patient 1	Patient 2	Patient 3	Patient 4
	26	31	34	39
CL estimates from CR model	0.048	0.057	0.086	0.123
	[0.047–0.049]	[0.056–0.058]	[0.085–0.088]	[0.120–0.125]
CL estimates from CYS model	0.039	0.047	0.096	0.128
	[0.038–0.040]	[0.046–0.048]	[0.095–0.098]	[0.125–0.130]

CL, vancomycin clearance (mean [95% confidence interval] in L/h*kg); CR model, final model based on Serum Creatinine; CYS model, alternative model based on Serum Cystatin C.

**FIGURE 1:**
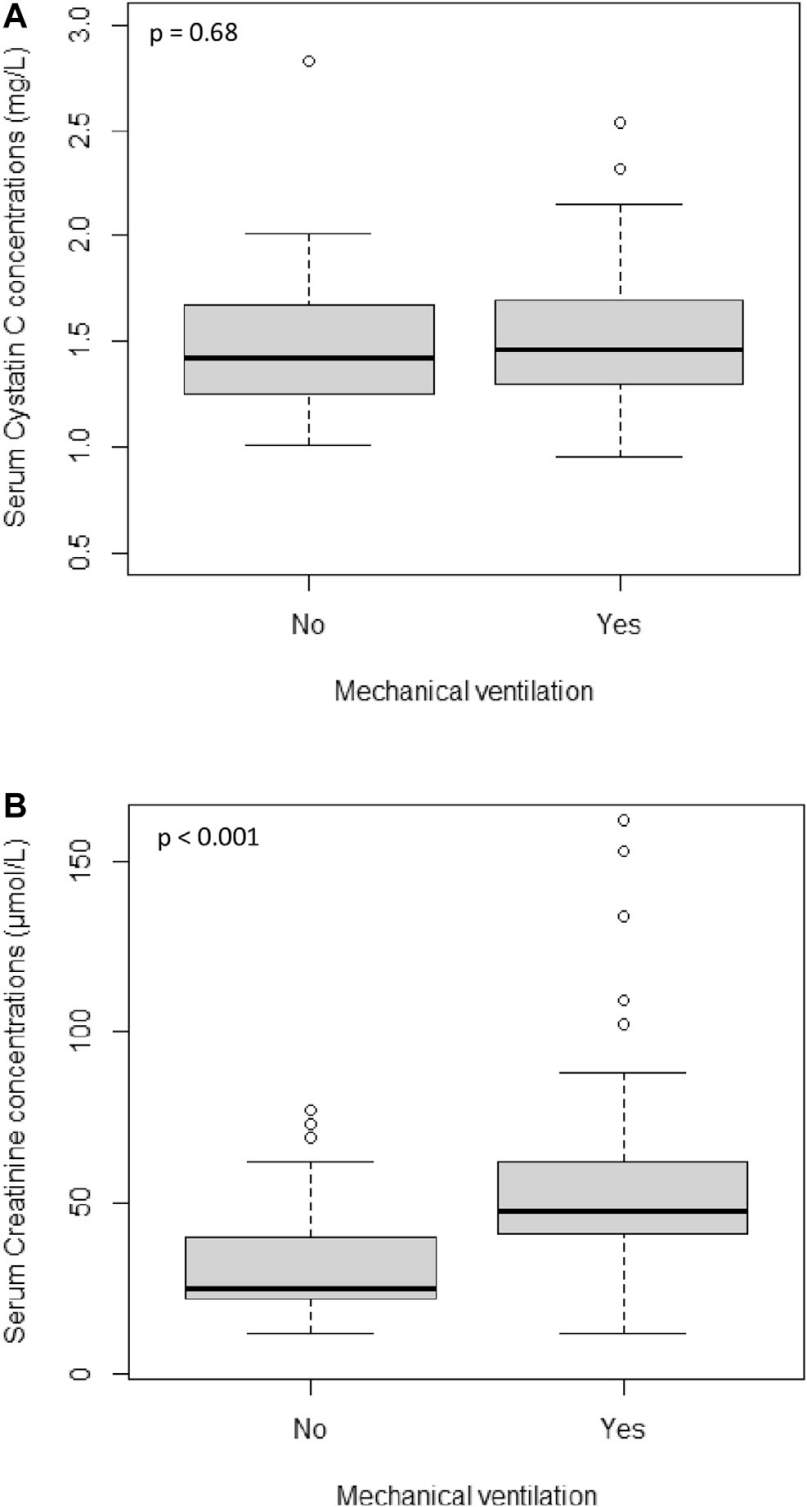
Distribution of Serum Cystatin C concentrations **(A)** and Serum Creatinine concentrations **(B)** in patients treated with and without mechanical ventilation.

Based on this finding, we further explored effects of mechanical ventilation on SCr and S-CysC. Figure 1 showed that SCr levels were higher in patients undergoing mechanical ventilation (median levels increasing from 25 to 48 µmol/l; *p* < 0.001) while S-CysC concentrations remained stable with and without mechanical ventilation (median levels of 1.46 and 1.43 mg/l, respectively; *p* = 0.68).

**FIGURE 2:**
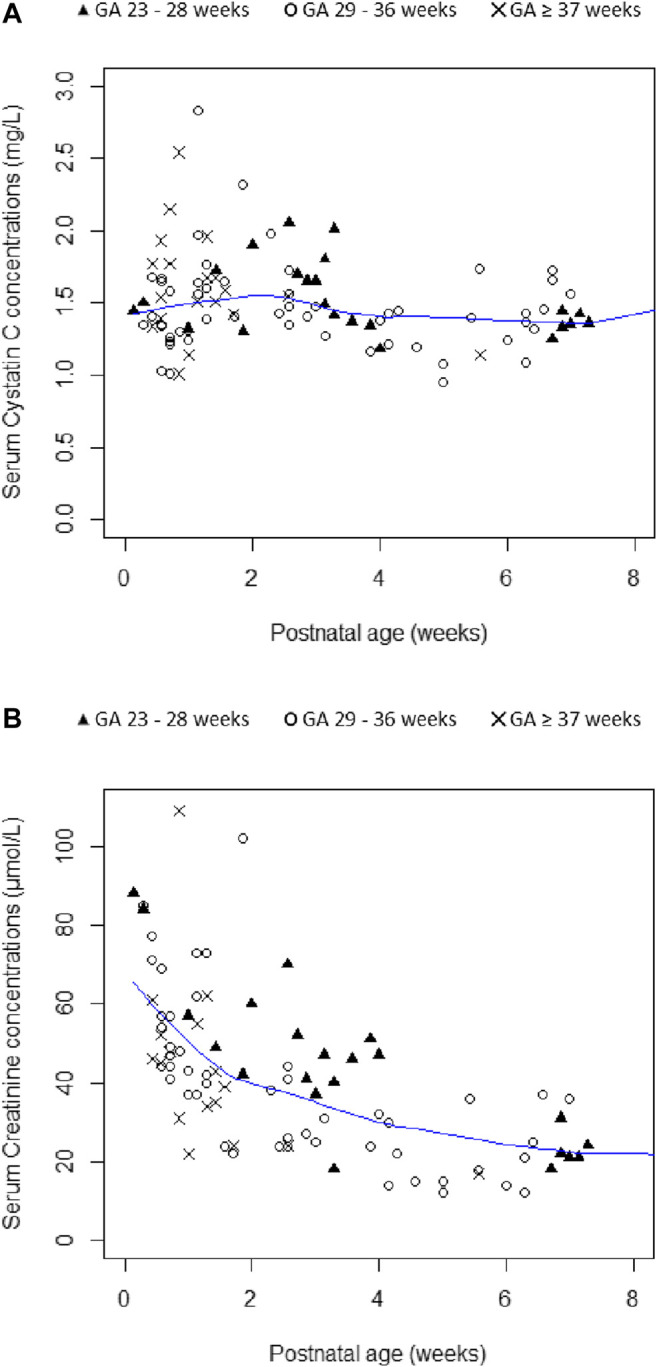
Plots of Serum Cystatin C concentrations **(A)** and Serum Creatinine concentrations **(B)** according to gestational age (GA) and postnatal age. Smooth lines are represented in blue color.

Additionally, as shown in Figure 2, regardless of GA group, SCr levels decreased gradually with PNA during the first two months of life while S-CysC concentrations remained relatively stable over the same time period. Then, S-CysC levels were significantly lower in patients treated with aminoglycosides (median levels decreasing from 1.56 to 1.35 mg/l; *p* < 0.001) while SCr concentrations were not impacted by aminoglycosides exposure (*p* = 0.73).

## Discussion

In the present study, a population PK analysis was performed to compare SCr and S-CysC as covariates on vancomycin CL in neonates undergoing intensive care treatment. The inclusion of SCr into the allometric model reduced IIVCL from 47 to 34%, while S-CysC reduced IIVCL from 47 to 40% (Table 2). Following stepwise covariate model building, SCr was retained as single marker of kidney function (after accounting for weight and PMA) in the final model. Compared to the final model based on SCr, the alternative model based on S-CysC showed very similar performance (e.g. BIC of 578.3 vs. 576.4) but included, besides the effects of size and maturation, the impact of mechanical ventilation as additional covariate on vancomycin CL.

Kidney function is the determining factor of dosing optimization for drugs predominantly eliminated by the kidney. Brou et al. recently reviewed the available PK studies comparing the impact of S-CysC and SCr on the CL of renally eliminated drugs (Brou et al., 2015). Among the 14 studies identified, only one was conducted in children, with the youngest patient being 4 years of age (Halacova et al., 2008). The present study is to our knowledge the first population PK study comparing S-CysC and SCr as covariates on drug CL in neonates undergoing intensive care treatment.

SCr and S-CysC concentrations measured in this study were in the range of reference values previously reported in neonates (Finney et al., 2000; Allegaert et al., 2015; Wilhelm-Bals et al., 2019). Given available data, our covariate analysis led to retain SCr rather than S-CysC as single marker of kidney function in the final vancomycin PK model. Our results are in agreement with recent findings of Wilhelm-Bals et al. showing that inulin CL correlated with SCr but not with S-CysC in a neonatal population with similar ranges of GA (26–40 weeks vs. 23–41 weeks in our population), S-CysC concentrations (0.87–2.4 mg/l vs. 0.95–2.8 mg/l in our population) and SCr concentrations (28–163 µmol/l vs. 12.0–153 µmol/l in our population) (Wilhelm-Bals et al., 2019).

The behavior of SCr and S-CysC levels in our population is in agreement with previous findings from the available literature. First, in the present study, SCr was shown to be impacted by PNA while S-CysC concentrations were stable during the first two months of life. In agreement with previous reports, SCr decreased gradually with PNA (Figure 2) because of residual maternal creatinine and tubular reabsorption by the immature kidney in early life (Abitbol et al., 2014; Kastl, 2017). Whatever differences in post-natal profile of SCr and S-CysC concentrations, antenatal and postnatal kidney maturation is reflected by PMA, a significant covariate on vancomycin CL in both CR and CYS models. Second, in accordance with previous findings by Abitbol et al. (2014), aminoglycosides exposure was associated with lower S-CysC values in our population. As previously suggested, this could be due to a competition between S-CysC and aminoglycosides both being ligands of the megalin receptor in the proximal tubule (Konopska et al., 2013). In other words, S-CysC concentration may be lowered in the presence of aminoglycosides independent of underlying kidney function. Clinicians should be aware of this potential confounding factor to interpret S-CysC for kidney assessment.

In this study, the inclusion of SCr into the allometric model reduced IIVCL from 47 to 34% while S-CysC reduced IIVCL from 47% to only 40% (Table 2). The comparison of CR and CYS models leads to the hypothesis that mechanical ventilation could at least partly explain this finding. Our results showed that the portion of IIVCL dependent on the effect of mechanical ventilation on kidney function was better reflected by SCr than by S-CysC. A 30% lower vancomycin CL was shown in neonates undergoing mechanical ventilation (factor 0.69 in CYS model). This is consistent with previous data from Perkins et al. showing that mechanical ventilation is a well-documented cause of decrease in cardiac output, hepatic and renal blood flow, GFR, and urine flow (Perkins et al., 1989). Vancomycin CL may be reduced in patients undergoing mechanical ventilation because of decreasing renal blood flow or GFR. Interestingly, the decrease in vancomycin CL due to mechanical ventilation stayed significant after integration of other covariates in the CYS model but not in the CR model. Indeed, accounting for mechanical ventilation further improved predictive performance of the CYS model (causing a further significant drop of 14 units in the OFV and a concomitant reduction in IIVCL from 36.1 to 30.3%) but did not significantly improve CR model (Table 2). This may suggest that S-CysC is less sensitive than SCr to the changes of GFR due to mechanical ventilation (Figure 1). Additional studies are needed to further explore this hypothesis.

As recently highlighted by the review of Muhari-Stark et al., currently available GFR-estimating formulas raise issues for neonatal use (Muhari-Stark and Burckart, 2018). Only two of the SCr-based GFR-estimates developed for paediatric use were derived from data in preterm and term neonates (Brion et al., 1986; Wilhelm-Bals et al., 2019). Their applicability is limited by the fact that SCr values were determined using the Jaffe method rather than the enzymatic one. None of the S-CysC-based GFR-estimates reported to date has been validated in neonates. In our population, none of the three tested GFR-estimates was superior to SCr alone as covariate on vancomycin CL (Table 2). We were not able to test GFR-estimates based on height as height was not available in all study subjects.

The vancomycin CL estimates based on both CR and CYS models are in accordance with values previously reported (de Hoog et al., 2004; Jacqz-Aigrain et al., 2019). Although comparison of BIC values cannot be interpreted statistically, a drop of 2 is often admitted as a threshold for considering one model over another (Mould and Upton, 2013). As shown by the ∆BIC of 1.9 between CYS and CR models, the use of S-CysC rather than SCr as single marker of kidney function provided a similar fit of our vancomycin data. However, for similar performance, CYS model required the inclusion of one additional covariate compared to CR model, which was only based on CW, PMA, and SCr. The pragmatic implications of these findings have to be considered for neonatal clinical practice. Due to the wide IIVCL shown in neonates, vancomycin dosage individualization is imperative. However, especially in NICUs, vancomycin treatment is frequently urgently needed, requiring simple, “easy to use” dosing recommendations. This is also a way to minimize prescriptions errors. Thus, leading to similar goodness of fit, the simplest model (CR model) might be preferred for implementation in clinical practice.

A limitation of our study is the sparse PK sampling. However, as reflected by the satisfying relative standard error of parameter estimates (i.e. <30% for CLTV, Effage, Effkidney and Effventilation—Tables 3 and 4) and other above-mentioned parameters of goodness of fit, this is expected to have limited impact on the primary objective of drug CL estimation. Second, both SCr and S-CysC levels might be affected by the assays used in this study. Different methods were previously reported to quantify these markers with between assay differences (Allegaert et al., 2015; Wilhelm-Bals et al., 2019). Finally, further investigations are needed to explore more in depth the pathophysiological differences between SCr and S-CysC, especially in patients undergoing mechanical ventilation.

## Conclusion

Population PK analysis led to retain SCr as single marker of kidney function to estimate vancomycin CL in neonates undergoing intensive care treatment. Compared to the final model based on SCr, the alternative model based on S-CysC showed very similar performance but included the effect of mechanical ventilation on vancomycin CL, as additional covariates to S-CysC, CW and PMA. SCr and S-CysC are both relevant renal markers for individualization of vancomycin dosing in critically ill neonates. However, if using S-CysC for this purpose mechanical ventilation needs to be taken into account.

## Data Availability

The raw data supporting the conclusion of this article will be made available by the authors, without undue reservation.
