# Supplementary material for: Serum Creatinine and Serum Cystatin C are Both Relevant Renal Markers to Estimate Vancomycin Clearance in Critically Ill Neonates
Source: Front Pharmacol. 2021 Mar 19;12:634686. doi: 10.3389/fphar.2021.634686 (PMC8104087; doi:10.3389/fphar.2021.634686)
Supplement: Supplementary file 1 [file datasheet1.pdf]

# *Supplemental material*

**Figure S1** Plot of vancomycin concentrations versus time

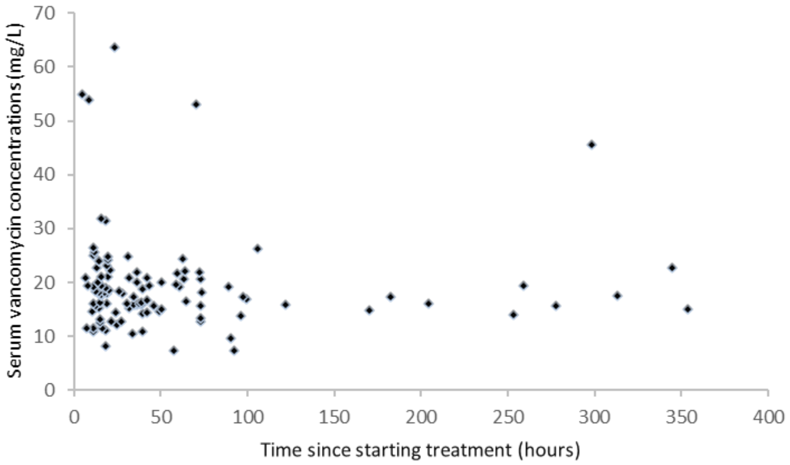

**Figure S2** Evaluation of the final model based on Serum Creatinine (CR model). (A) Population predicted (PRED) versus observed concentrations (DV); (B) individual predicted (IPRED) versus DV; (C) conditional weighted residuals (CWRES) versus time; (D) CWRES versus PRED; (E) QQ-plot of the distribution of the normalized prediction distribution errors (NPDE) versus the theoretical N(0,1) distribution; (F) histrogram of the distribution of the NPDE, with the density of the standard Gaussian distribution overlaid.

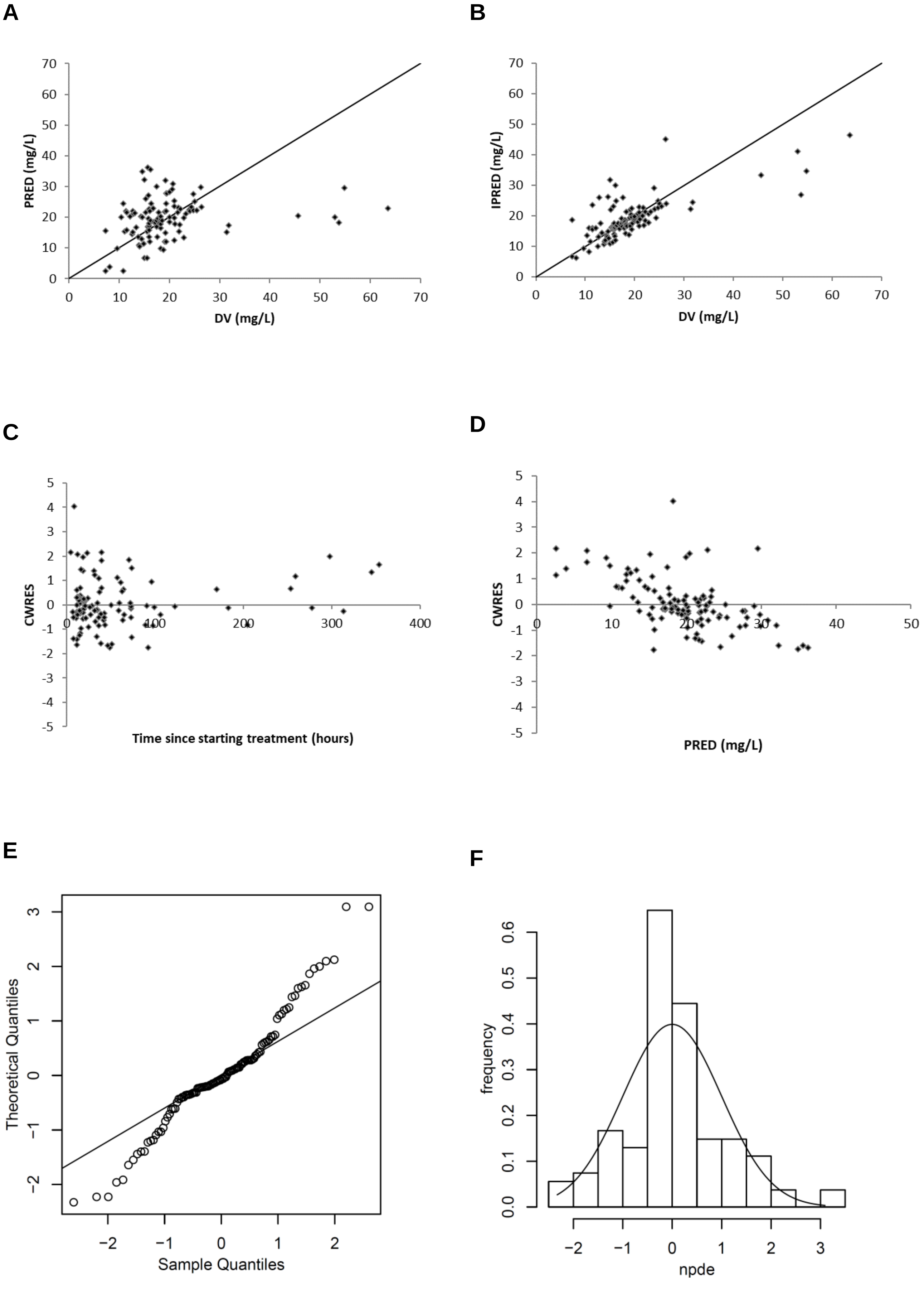
A
PRED (mg/L)
DV (mg/L)
B
IPRED (mg/L)
DV (mg/L)
C
CWRES
Time since starting treatment (hours)
D
CWRES
PRED (mg/L)
E
Theoretical Quantiles
Sample Quantiles
F
frequency
npde

**Figure S3** Evaluation of the alternative model based on Serum Cystatin C (CYS model). (A) Population predicted (PRED) versus observed concentrations (DV); (B) individual predicted (IPRED) versus DV; (C) conditional weighted residuals (CWRES) versus time; (D) CWRES versus PRED; (E) QQ-plot of the distribution of the normalized prediction distribution errors (NPDE) versus the theoretical N(0,1) distribution; (F) histrogram of the distribution of the NPDE, with the density of the standard Gaussian distribution overlaid.

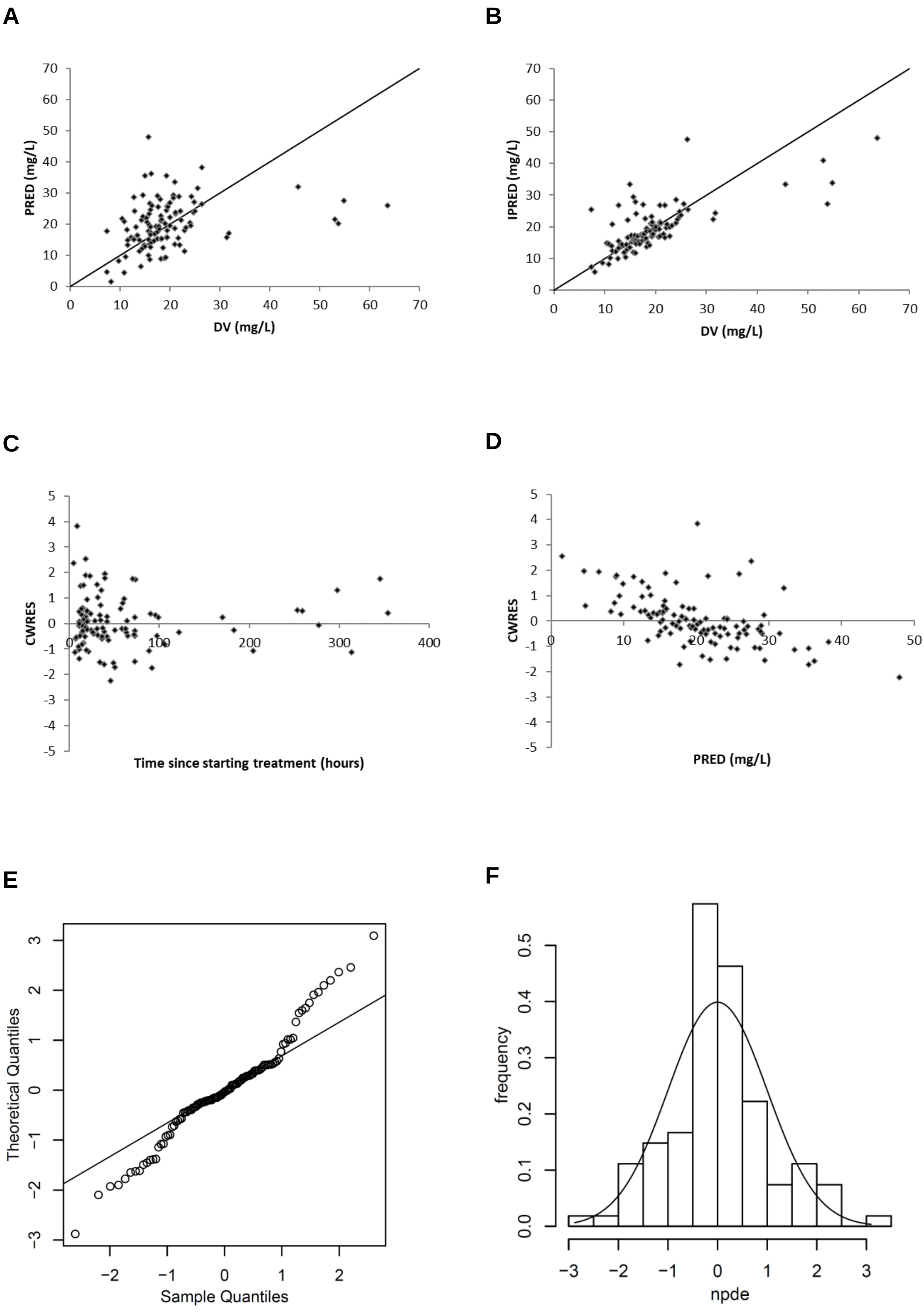
A
PRED (mg/L)
DV (mg/L)
B
IPRED (mg/L)
DV (mg/L)
C
CWRES
Time since starting treatment (hours)
D
CWRES
PRED (mg/L)
E
Theoretical Quantiles
Sample Quantiles
F
frequency
npde

frontiers
